# How context affects transdisciplinary research: insights from Asia, Africa and Latin America

**DOI:** 10.1007/s11625-022-01201-3

**Published:** 2022-08-23

**Authors:** Flurina Schneider, Aymara Llanque-Zonta, Onintsoa Ravaka Andriamihaja, R. Ntsiva N. Andriatsitohaina, Aung Myin Tun, Kiteme Boniface, Johanna Jacobi, Enrico Celio, Clara Léonie Diebold, Laby Patrick, Phokham Latthachack, Jorge Claudio Llopis, Lara Lundsgaard-Hansen, Peter Messerli, Stellah Mukhovi, Nwe Nwe Tun, Zo Hasina Rabemananjara, Bruno Salomon Ramamonjisoa, Sithong Thongmanivong, Thoumthone Vongvisouk, Daovorn Thongphanh, Win Myint, Julie Gwendolin Zaehringer

**Affiliations:** 1 Centre for Development and Environment (CDE), University of Bern, Mittelstrasse 43, 3012 Bern, Switzerland; 2 Institute for Spatial and Landscape Development IRL, Planning of Landscape and Urban Systems PLUS, ETH Zürich, Stefano-Franscini-Platz 5, 8093 Zurich, Switzerland; 3 Environmental Care and Community Security Institution ECCSi, 108, 2nd Flr, San Chaung St., Shin Saw Pu Ward, Sanchaung, Yangon, Myanmar; 4 Ecole Supérieure des Sciences Agronomiques, Département des Eaux et Forêts, Université d’Antananarivo, B.P 175-101, Antananarivo, Madagascar; 5 Faculty of Forest Science, National University of Laos, P.O. Box 7322, Vientiane, Laos; 6 School of Natural Sciences, Bangor University, Bangor, LL57 2UW UK; 7 Institute for Social-Ecological Research (ISOE), Hamburger Alee 45, 60486 Frankfurt, Germany; 8 Faculty of Biosciences, Goethe University Frankfurt, Max-von-Laue-Str. 9, 60438 Frankfurt, Germany; 9 Department of Geography and Environmental Studies, University of Nairobi, P.O. Box 30197-00100, Nairobi, Kenya; 10 Faculty of Sustainability, Leuphana University, Universitätsallee 1, 21335 Lüneburg, Germany; 11 Centre for Training and Integrated Research in ASAL Development (CDTRAD), P.O. Box 144-10400, Nanyuki, Kenya; 12 Senckenberg Biodiversity and Climate Research Centre SBiK-F, Georg-Voigt-Straße 14, 60325 Frankfurt, Germany

**Keywords:** Transdisciplinarity, Context, Global South, Epistemology

## Abstract

Transdisciplinary research (TDR) has been developed to generate knowledge that effectively fosters the capabilities of various societal actors to realize sustainability transformations. The development of TDR theories, principles, and methods has been largely governed by researchers from the global North and has reflected their contextual conditions. To enable more context-sensitive TDR framing, we sought to identify which contextual characteristics affect the design and implementation of TDR in six case studies in Asia, Latin America, and Africa, and what this means for TDR as a scientific approach. To this end, we distinguished four TDR process elements and identified several associated context dimensions that appeared to influence them. Our analysis showed that contextual characteristics prevalent in many Southern research sites—such as highly volatile socio-political situations and relatively weak support infrastructure—can make TDR a challenging endeavour. However, we also observed a high degree of variation in the contextual characteristics of our sites in the global South, including regarding group deliberation, research freedom, and dominant perceptions of the appropriate relationship between science, society, and policy. We argue that TDR in these contexts requires pragmatic adaptations as well as more fundamental reflection on underlying epistemological concepts around what it means to conduct “good science”, as certain contextual characteristics may influence core epistemological values of TDR.

**Supplementary Information:**

The online version contains supplementary material available at 10.1007/s11625-022-01201-3.

## Introduction

In addition to rising calls for fundamental, social–ecological transformations to preserve life on Earth and advance towards sustainability, a number of scholars have begun to emphasize the need for transformation of our corresponding science systems (Care et al. 2021; Fazey et al. 2020; Moore et al. 2017). For the most part, sustainability science has so far been successful in describing and explaining the current state of *unsustainable* development, including biodiversity loss, food insecurity, and socio-economic inequalities. However, it has struggled to generate knowledge that effectively fosters the capabilities of diverse societal actors to implement necessary changes. Knowledge co-production approaches such as transdisciplinary research (TDR) were explicitly developed for this purpose.

In sustainability science, TDR is understood as a research approach that tackles “real world” societal problems (Klein 2001, p. 4) by means of interdisciplinary collaboration and interactions with societal actors (Jahn et al. 2012). To support its operationalization, ideal–typical models, methodologies, and design principles have been developed (Jahn and Keil 2015; Lang et al. 2012; Scholz and Steiner 2015). The ideal–typical models often distinguish between three overlapping phases where researchers and societal actors collaboratively frame the problem and goals, co-produce solution-oriented knowledge, and jointly explore pathways to impact. While these three phases constitute the ‘ideal’ way of implementing TDR, in reality, it is much more complex. Design principles address issues related to the inclusion of all relevant societal actors and perspectives, facilitation of high-quality collaboration processes on equal ground, and joint reflection on the different viewpoints and interests. TDR approaches have been applied in both the global North and South. Recently, however, Southern scholars such as Berger-Gonzales et al. (2016) and Van Breda and Swilling (2019) have questioned the one-to-one applicability of what they see as a “Western” TDR concept. They point to considerable differences in the socio-economic, political, and historical contexts of many Southern countries, including volatile political and economic circumstances, greater informality, and distinct norms regarding social interaction. From their perspective, the current ideal–typical TDR models and design principles are too standardized and not flexible enough to respond to these dynamic contextual conditions.

In this article, we aim at critically examining TDR experiences in six different contexts in the global South. In the following section, we first scrutinize the significance of “context” in TDR conceptualizations in the global North and South. We then present empirical research showing how context conditions influenced TDR while engaging with them. Finally, we end the article by reflecting on what this might mean for TDR as a scientific approach.

### TDR and the importance of context

Context is a key concept in TDR, but rarely explicitly defined and theorised. Context is generally understood as the social, political, and environmental settings in which the investigated “real world” sustainability problems manifest (Carew and Wickson 2010; Ott and Kiteme 2016; Sim et al. 2019; van Dijk 2007). In some cases, context references also include the *researcher’s* context—including existing power asymmetries at research institutions, accepted epistemologies, and available funding (Carew and Wickson 2010; Simon et al. 2020b; Simon 2021). TDR scholars stress the need for researchers to engage deeply with their problem contexts to generate the knowledge needed to effectively tackle sustainability problems. This is seen as crucial independently of where the research takes place (e.g. in the researcher’s neighbourhood, a different ethnic region of his/her own country or in a foreign country). As a consequence, TDR outcomes are conceived of as producing insights that are applicable to specific contexts, rather than striving for generalizable findings (Adler et al. 2018). Two different arguments are used to justify this claim (Pohl and Hirsch Hadorn 2007):

1. *Contextuality is required to grasp the meaning of sustainability*: Sustainability is an inherently normative concept encompassing various—often conflicting—values regarding how a more desirable future should look like (Schneider and Rist 2014; Wiesmann et al. 2011). As value statements require deliberations among involved societal actors, the act of identifying a sustainability problem implies engaging with the relevant actors in a specific problem context. These arguments are rooted in several schools of thought, including “post-normal” science (Funtowicz and Ravetz 1993).
2. *Contextuality is required to generate actionable knowledge:* Only when focusing on a specific problem context—with its existing needs, technologies, regulations, and power relations—can knowledge be generated that is capable of being applied and bringing about desired changes (Carew and Wickson 2010). This argument was fostered by debates about knowledge production in the context of its application, in particular with reference to “mode 2” knowledge production. This approach holds that theory and practice cannot be separated from their context of production and that inclusion of various forms of expertise, including lay perspectives, is needed to foster “socially robust knowledge” (Gibbons et al. 1994; Nowotny et al. 2001).

While context is a relevant concept for any research, e.g. for justifying case study selection and explanations for variations in findings, in TDR for sustainability, the explicit involvement of the value and action dimensions bring up additional aspects. Scholars from social sciences (Dilley 1999, 2002; van Dijk 2007; Lawson 2006) and programme evaluation (Chouinard and Milley 2016) stress that researchers in such fields must be aware of the double character of context: context is “constitutive of social action and itself the outcome of social action” (Dilley 2002, p. 445). Hence, understanding TDR as social action, it is at the same time influenced by and influencing context. In other words, contexts are not just pre-given, external conditions, but reconstructed by interpretations of and social interactions between researchers and local actors (Dilley 2002; Chouinard 2016).

TDR scholarship considers both aspects. On the one hand, it gives high importance to the reconstruction process: joint problem (context) framing with all relevant actors is seen as a first step of any TDR project, followed by joint knowledge production and exploring pathways to impact. On the other hand, it highlights the performative dimensions of context by stressing the need to adapt to the local circumstances, which appear difficult to change in a single project. Relatedly, Lang et al. (2012) have stated that ideal–typical TDR models “should not be understood as ‘a recipe’ applicable in any given context…[rather,] the compiled principles need to be specifically adapted for each project”. As a result, stakeholder interaction processes must be tailored to the specific contextual conditions, such as dialogue cultures and literacy levels (Schneider and Buser 2018). Better understanding of the implications of different contextual conditions for TDR is seen as an important research gap (Lang et al. 2012; Schneider and Buser 2018). Therefore, our paper focuses on the performative dimension of context.

### TDR in contexts of the global North and South

In connection with its use and application in sustainability science, TDR has been practiced and reflected upon in various contexts of the global North and South (e.g. Lang et al. 2012; Rist et al. 2011; Wiesmann et al. 2011; Vienni Baptista et al. 2019). This has given rise to a rich terrain of different approaches and debates. On the one hand, the resulting diverse approaches can be attributed to distinct problem constellations manifesting in specific contexts. On the other hand, they are rooted in distinct conceptions of what constitutes good science as well as how science, society, and policy should interrelate.

TDR approaches developed in the global North tend to lean heavily on its history of political and scientific thought. This includes reference to concepts of Western democracy and related principles of open discourse, value deliberation, and equal participation of all citizens, for example, as described in the scholarship of Jürgen Habermas (1981). Some authors explicitly argue for the democratization of knowledge production as a goal in itself (Ott and Kiteme 2016), whereas others refer to democracy-related principles when justifying the “scientificality” of TDR (see Sect. 1.1). Northern approaches also often include references to epistemological ideals of good science, such as “rigour” and “independence” (Scholz and Steiner 2015; van Kerkhoff 2014), and provide considerations on the appropriate relationship between “facts” and “values”. Further, significant emphasis is frequently placed on positioning TDR as a theoretically well-founded form of research that follows accepted epistemological standards. As a consequence, many TDR approaches developed in the global North emphasize standardized processes (Scholz and Steiner 2015), generic principles (Lang et al. 2012), and quality criteria (Jahn et al. 2012).

TDR literature from the global South is partly rooted in different strands of thinking. For example, many writings from Latin America highlight the need for valuing indigenous knowledge (Rist et al. 2011) and deconstructing epistemic power structures (Alonso-Yanez et al. 2019). Referring to the thinking and practices behind Paulo Freire’s “pedagogy of the oppressed” (Freire 1973) and Leonardo Boff’s “liberation theology”, they also often stress the need for the creation of critical consciousness, liberation of the oppressed, and equity-oriented partnerships between local people, researchers, and other societal actors. This may involve efforts towards decentering academic concepts and practices to create space for other knowledge communities (Alonso-Yanez et al. 2019). In addition, development visions based on “modernization” concepts are often criticized (Hirsch Hadorn et al. 2006). African and Asian scholarship often refers to TDR concepts developed in the global North, but frequently recommends specific adaptations. For example, some African scholars particularly emphasize the need for capacity building, emergence, or transformation (ISC 2019; Ott and Kiteme 2016; van Breda and Swilling 2019), whereas Asian scholars emphasize the challenges of doing TDR under authoritarian and hierarchical governance structures (Siew et al. 2016; Sim et al. 2019).

Nevertheless, despite the existing richness of approaches, the overall scientific discourse regarding TDR theories, principles, and methods has largely been steered by researchers of the global North and reflects experiences of the global North (Chammas et al. 2020; Ely et al. 2020; Siew et al. 2016).

### Excessive standardization versus everything goes in TDR

Several authors have praised the growing diversity of TDR approaches and concepts. These voices became stronger in the recent debate over “decolonization of science”, which argues that the hegemony of Western thought and culture needs to be dismantled to make way for local philosophy, traditions, and forms of knowledge production (Nordling 2018). At the same time, there has been intense debate over (lacking) agreement on a joint definition of TDR. For example, Jahn et al. (2012) highlight the risks of rhetorical mainstreaming of TDR in Europe, where the term is increasingly used without reference to the scholarly debate. They argue that finding common ground on a general definition of TDR is important to ensure the quality standards needed to enable further establishment of TDR in academia. In the view of the paper authors, both of these arguments have value. TDR concepts that are vague and ambiguous pose risks to scientific quality and comparability, as “everything is possible” (Jahn et al. 2012), but those that are too narrow may lack contextualization, devalue local scientific traditions (Ely et al. 2020) or pose risk of reproducing “dominant Euro-Western paradigms” and “epistemological imperialism” (Chouinard 2016). Overall, charting a path forward out of these emerging tensions is not an easy task (Berger-González et al. 2016; Steelman et al. 2015). With the present article, we seek to advance the development of a context-sensitive TDR understanding by identifying the contextual characteristics that influenced TDR in six different global South settings, and their implications for TDR as a scientific approach.

## Methodology

### Case studies

Our analyses draw on six case studies: two in Asia (Myanmar and Laos), two in Africa (Kenya and Madagascar), and two in Latin America (Bolivia and Brazil). The sites and case studies belong to two TDR projects funded by the Swiss National Science Foundation (SNSF) and the Swiss Agency for Development and Cooperation (SDC) within the “Swiss Programme for Research on Global Issues for Development (r4d programme)”. The r4d programme supports research aimed at solving global problems with a focus on low- and middle-income countries. The two projects were launched in 2015 and received funding for 6 years. An overview of the respective project goals, institutional setup, and key TDR activities is presented in Table 1.

**Table 1:** Overview of the two TDR projects

	Telecoupling project	FoodSAF project
Title	Managing Telecoupled Landscapes for the Sustainable Provision of Ecosystem Services and Poverty Alleviation	Towards Food Sustainability: Reshaping the Coexistence of Different Food Systems in South America and Africa
Regions	Myanmar, Laos, and Madagascar	Bolivia, Brazil, and Kenya
Goal	Developing strategies for managing telecoupled landscapes for the sustainable provision of ecosystem services and poverty alleviation	Providing evidence-based scientific knowledge for the formulation and promotion of innovative strategies and policy options that improve individual and aggregate levels of food system sustainability
Institutional setup	Project coordination in Switzerland; three thematic work packages; partnerships with one research institution per case study country leading the local implementation of work packages; multi-stakeholder learning groups in each country; annual project meetings between all senior researchers and postdocs. A total of seven senior researchers; five postdocs; nine PhDs, and various MSc students and research assistants	Project coordination in Switzerland; four thematic work packages and one integrative work package; partnership with one research/implementation institution each in Bolivia and Kenya, who co-coordinate the project, co-supervise theses, and subcontract others. In the second phase (2018–2020), the project expanded to include Colombia, Brazil, Ghana, and Zambia, with one main partner in each country
Overall TDR philosophy	Rather Western-based TDR philosophy	Rather Latin American-based TDR philosophy
Key TDR activities	Inception phase: establishment of partnerships between local and foreign researchers and societal actors; co-design of research foci and case selection Empirical research phase: researchers of different disciplines conduct their studies (e.g. participatory mapping, interviews, surveys, focus groups); multi-stakeholder learning groups; regular exchanges within and between work packages and countries Synthesis phase: integration of insights within and across different case study countries Transformative action phase: based on research insights, transformative action projects designed and implemented together with societal actors	A) Preparation phase with one workshop each in Kenya and Bolivia, with diverse partners on hand to identify central food system problems and elaborate joint ideas for the proposal B) First project phase consisting of empirical research (e.g. surveys, livelihoods assessments, environmental footprint analysis), partly including transdisciplinary research methods (e.g. focus groups and workshops for compiling and sharing recipes linked to food traditions and crop diversity) C) Second project phase comprising application of the FoodSAF tool to identify core problems of food systems develop collective actions, and implement the latter
Disciplines	Geography, biology, environmental sciences, economics, agriculture and forestry management, land science, landscape development, political sciences	Geography, law, social anthropology, human rights, agronomy, environmental sciences, economics
Societal actors	Smallholders, cash crop producers and exporters, government at all levels, environmental NGOs, civil society organizations	Pastoralists, smallholders, large-scale farmers, agro-industrial companies, transporters, logistics companies, processors, retailers, consumers, government, cooperatives, social movements, farmer organizations, NGOS

### Northern Tanintharyi Region in southern Myanmar

This mountainous region is characterized by a humid tropical climate and a mix of competing land uses, including subsistence agriculture, commercial plantations (rubber, oil palm), forests, gas pipelines, and a planned “special economic zone”. The local population are mostly farmers and belong to different ethnic groups and religions (mainly Buddhist and Christians). The region was heavily affected by the civil war during the previous military government. In 2011/2012, a (temporary) transition to a semi-civil government was initiated. To date, both Myanmar’s government and local rebel groups claim (competing) sovereignty over parts of the area. Myanmar is categorized as lower-middle income.

### Luang Namtha province in northwestern Laos

This mountainous province features a humid tropical climate, a variety of agricultural land uses, as well as the Nam Ha National Protected Area. The local population are mostly farmers and belong to different ethnic groups, including Lao Lum, Khmu, Mien, Hmong, and Akha. Traditionally, they depended on subsistence farming, particularly upland rice cultivation in shifting cultivation systems. More recently, they have become involved in cash crop production for markets in China (e.g. banana, rubber, sugarcane) as a consequence of Lao PDR’s new export-oriented economic policy and China’s opium replacement policy. Lao PDR is a one-party socialist republic and its economy is categorized as lower-middle income.

### Maroantsetra District in northeastern Madagascar

This district is characterized by a humid tropical climate and small patches of cropland. Its population mostly belongs to the Betsimisaraka ethnic group, with local livelihoods depending on subsistence farming (paddy and upland rice growing in shifting cultivation systems) as well as cash crop cultivation (vanilla, cloves). The district is surrounded by two large protected areas that are aimed at not only protecting local biodiversity-rich forests, but also restrict land access for farmers. Growing interest in cash crop cultivation has exacerbated conflicts of interest between land users. Madagascar is a democracy and is categorized as low income.

### Northwest Mt Kenya region in Kenya

This region is located at the slopes of Mount Kenya. It features diverse agroecological areas and a variety of food systems, including agro-industrial-based systems producing vegetables for export to European markets, as well as pastoralists, ranchers, and smallholder-based systems producing beef, milk, and wheat for the national market; and finally smallholder-based systems producing food for local markets. Smallholders participating in contract-farming schemes for export have experienced difficulties in meeting the standards set by the EU and supermarkets, e.g. regarding the intensive use of pesticides. Kenya is a representative democracy and is categorized as lower-middle income.

### Sucre municipality and Samaipata municipality in Bolivia

Sucre and Samaipata are two cities located in the Bolivian Andes in a semi-arid climate. Sucre is the capital of Bolivia with over 200,000 inhabitants; Samaipata a touristic centre with less than 5000 inhabitants. Sucre is experiencing steady growth from rural–urban migrants, many of whom carry on their agricultural activities in the city, whether in their backyard or on their rooftop. Over 70% of such urban farmers are women. Samaipata has developed various concepts for ecotourism and agroecology. Bolivia is a representative democracy with an indigenous government since recently and is categorized as lower–middle income.

### State of Santa Catarina in southern Brazil

Seara Municipality in the western part of Santa Catarina has a humid subtropical climate. Descendants of Italian and German migrants here practice diversified family farming. However, many local farmers are under contracts with JBS, the world’s largest meat company, which limits them to raising only one type of animal. Various local farmers, consumers, research organizations, NGOs and organized movements such as “Slow Food” have united to promote food traditions linked with healthy agroecosystems and sustainable, diversified farms. At the same time, food safety rules have prohibited family farmers from selling traditional products such as raw milk cheese. Brazil is a democracy and is categorized as upper–middle income.

### Methods

Our research is guided by a “reflexive model of science” (Burawoy 1998), which promotes active engagement with the object of analysis and study participants in the course of knowledge generation. In this mode of research, “reflective practitioners” (Schön 1983) apply methods of self-reflection and formative assessment (Lang et al. 2012; Schneider et al. 2019). It is a highly suited mode of research for our research question, as the research team is very knowledgeable about the investigated practices and intrinsically motivated to examine them critically to become more effective in applying TDR approaches in various contexts. A potential disadvantage of the approach is a possible blindness to alternative interpretations.

All of the co-authors were involved in one of the two TDR projects and worked in 1–3 case study contexts, whether in designing or implementing the TDR approach or in both. Senior researchers from both projects were experienced in conducting and studying TDR. They organized regular debriefing sessions and annual workshops to reflect on TDR processes over the lifetime of the two projects. In a first step—by comparing their different TDR approaches and insights—a core team of senior researchers identified four TDR *process elements* that were relevant in both projects/all six contexts and appeared to be influenced by the different contexts, namely (1) coming together, (2) interacting, (3) co-producing knowledge, and (4) exploring paths to transformation (for more details, see Sect. 3.1). These four process elements are not independent, but rather build on one another. For example, co-producing knowledge involves characteristics that might be less relevant to interacting, considered on its own, but anything that is important to coming together and interacting is also relevant to co-producing knowledge. Second, by further exploring how the different contextual characteristics influenced the four TDR process elements, the participating researchers identified several *constituting context dimensions*. Identification of TDR process elements and constituting context dimensions was an iterative process. Third, the main author organized a structured online group discussion with each case study team, discussing the TDR process elements and context dimensions. In parallel, all participants recorded their experiences and knowledge in a shared google.doc table. The collected information was then standardized by the main author in several consultation rounds with the case study teams to harmonize the findings and make them comparable (Annex A). Fourth, based on the information collected, the main author conducted a comparative content analysis (Flick 2005), first per context dimension and later per process element. This analysis was critically scrutinized and complemented by all co-authors.

## Results

In the following, we present each TDR process element and associated context dimension that was identified through our research. Table 2 offers an overview of the concepts used. More details about the six case studies are provided in Annex A.

**Table 2:** Context dimensions affecting TDR process elements

TDR process element	Context dimension	What it is about
Coming together	Socio-political norms	Formal and informal rules to be followed when approaching actors for TDR
	Infrastructure	Availability and quality of transport and communication infrastructure
	Safety situation	Safety risks due to environmental conditions and violence
Interacting	Openness for interaction	Actors’ openness, willingness, and interest to engage in dialogue with researchers and with each other
	Languages	Linguistic competences, multilingualism, and communication styles
	Dialogue cultures	Communication patterns in group settings, role of deliberation in opinion formation, and mechanisms for creating shared understandings
Co-producing knowledge	Research institutions	Accepted practices within universities and research institutions; epistemologies, hierarchies
	Science–policy–society relations	Dominant perceptions of the proper relationship between science, policy, and society
	Hierarchies of knowledge holders	Accepted knowledge holders
	Literacy and learning habits	Literacy and forms of knowledge production and learning in everyday life
	Desirability of new knowledge	The value attached to new knowledge
Exploring paths to transformation	Concepts of development and the good life	Dominant ideas about development and the good life that shape people’s worldviews
	Development dynamics	Current development/transformation dynamics shaping the room for manoeuvre
	Contestation	Each issue’s level of contestation
	Actor agency	The degree of agency (interest and power to create change) that is held by those with a stake in the issue

### Coming together

Before starting any TDR project in a specific context, researchers and other societal actors—whether domestic or foreign—must physically come together and establish the ability to conduct TDR safely. Our research revealed that this requires becoming familiar with and managing different, sometimes challenging socio-political norms, infrastructure conditions, and safety situations.

*Socio-political norms*: Socio-political norms include the formal and informal rules to be followed when starting TDR. Formal rules involve written requirements codified in laws and agreements (e.g. official procedures to obtain travel and research permits); informal rules involve non-formalized norms including oral agreements, consent requests by powerholders, and tacit understandings about personal conduct in social interactions (e.g. expectations of etiquette). For example, in our Asian and African study sites, formal research permits issued by national authorities were mandatory, whereas in the Latin American cases official letters explaining the goals and benefits and/or agreements between institutions were required. In all cases, it was advisable to follow various informal rules, such as being accompanied by a locally respected person (“joint friends”), making courtesy visits, and reaching out to key organizations. Compliance with these norms was a precondition to establish good contacts with local actors, namely in cross-cultural settings, or where the concerned actors were in conflict (e.g. in Myanmar, where consent by the local rebel group was needed).

*Infrastructure:* This dimension involves the availability and condition of transport and communication infrastructure needed to come together, including technical means (roads, cars, motorbikes, boats, airplanes, fuel, internet, and phone coverage) as well as organizational and social issues, such as schedules, maintenance, reliability, comfort, road blockages, and time. The availability and conditions of infrastructure in our study locations were very diverse, for example, while the study sites in Brazil were generally comfortably accessible by car on good roads, some study sites in Madagascar could only be reached by a combination of boat, motorbike, and several hours of hiking. Overall, travel to all study sites was long and exhausting, e.g. due to mobility restrictions caused by weather, social unrest or lack of fuel. Some villages completely lacked internet and phone connections.

*Safety situation*: This dimension concerns risks of bodily harm. The risks may be related to environmental conditions such as tropical diseases (e.g. malaria, dengue, typhoid), cyclones, and floods; or they may be related to social conditions, such as poor medical care, frequent traffic accidents, or violence due to armed conflicts and criminality. In many of our case study sites, the teams had to deal with these types of challenging safety issues. Sometimes the challenges could be coped with through careful preparation and special arrangements (e.g. health prophylaxis, local guides, risk-management plans), but other times the project activities had to cease temporarily, on short notice, or could not even begin. For example, in Madagascar, the presence of illicit activities (rosewood logging/agricultural expansion in national parks) and related violence constrained fieldwork, and in Myanmar, some areas controlled by a local rebel group could not be visited.

### Interacting

TDR heavily depends on the quality of social interactions between various actors involved in the issue at stake. As a result, relationships of trust with various societal actors must be established to successfully conduct TDR. We found that this involves adapting TDR to societal actors’ openness for interaction (or lack thereof), language issues, and dialogue cultures.

*Openness for interaction*: This dimension involves the different societal actors’ willingness, and interest to engage in dialogue with researchers and with each other. In all of our case studies, some actors were very open, whereas others were reluctant or refused collaboration altogether. It largely depended on the actors’ expected and experienced benefits, the sensitivity of the researchers, as well as the history of research and/or conflict in the local setting. In some cases, local stakeholders could call to mind long histories of interethnic dispute, conflict, or “extractive research”. In this way, while all the research teams had to invest in trust building, their starting situation was very different from one setting to another. For example, the researchers in Kenya could build on a local research centre’s long-term partnership with societal actors in the area; in Madagascar, the researchers had to overcome research fatigue among villagers who were tired of being investigated due to prior research projects they perceived as extractive; and in Myanmar, the researchers sometimes experienced reservation among stakeholders who feared military surveillance. Importantly, people’s openness for interaction also changed over time. In all cases, the study subjects had to perceive the research as helpful in their daily struggles for them to continue to participate.

*Languages*: Language issues concern linguistic competences and communication styles required to communicate meaningfully with and among various societal actors. It involves the ability to speak, write, and understand each other, as well as body language and proper interpretation of meanings. Language challenges arose between local and foreign researchers, between researchers and local actors, and among various local actors—particularly in multi-ethnic settings and when (foreign) researchers did not speak the national or local languages. Challenges ranged from basic issues of understanding and comprehension of scientific concepts (e.g. ecosystem services, actor networks, future scenarios) to proper interpretation of the nuances of fundamental expressions like “yes” and “no” (e.g. in Asia saying “no” is considered impolite). While young men in the study sites were frequently able to understand the official national language, this was not always the case among young women and elders more broadly. Further, both the local actors and the researchers themselves felt uncomfortable if they were not fluent in the language of their interlocutor. Consequently, translation was regularly needed, but misunderstandings remained an issue.

*Dialogue cultures*: This dimension involves how people communicate in group settings, the role of deliberation in opinion formation, and mechanisms for creating shared understandings. There were noticeable differences between the dialogue cultures on display in the Asian, Latin American, and African study sites. In the Asian sites, the dialogues in workshop settings were often shaped by traditional hierarchical structures, in particular displays of deference to those considered of higher social rank, such as teachers, (male) elders, work-related superiors, and other authorities. During official discussions, lower ranked actors were hesitant to speak freely and tended to remain quiet. Women and young people often only spoke when directly questioned. Hierarchical structures also played a key role in the African study sites, but more actors—including women—were accustomed to speaking in group settings (e.g. based on storytelling culture) and different opinions were openly voiced and deliberated. Finally, in the Latin American sites, strong cultures of controversial political debate were exhibited. Possible hierarchies were scrutinized more openly and did not decisively impact joint decision-making.

### Co-producing knowledge

Co-production of knowledge is at the core of TDR. It refers to the process of generating novel knowledge while interacting with societal actors in a specific context. Our research showed that the space for co-production of new knowledge was shaped by the functioning of research institutions; dominant ideas about the relation between science, policy, and society; hierarchies of knowledge holders; literacy and learning habits; and the value attached to new knowledge.

*Research institutions:* This dimension concerns the functioning of research institutions, including the position of research, dominant epistemological values, and acceptable research practices. In all our case studies, the public universities involved were strongly focused on teaching. While in some countries (e.g. Brazil) research was also emphasized—including a highly developed academic culture and international networks—in other countries (e.g. Myanmar) research only played a marginal role, and corresponding quality criteria and links to the international research community were just being developed. However, funds for research were always scarce and academics were often burdened with administrative tasks and heavy teaching loads. Research opportunities often depended on access to international funding. In Laos, Kenya, and Madagascar, empirical fieldwork was the most common form of research; in Myanmar, deskwork and learning from teachers dominated; and in Bolivia and Brazil, critical approaches that emphasize reasoning and theory development were especially prevalent. In virtually all contexts, linking of theory and empirical work and interdisciplinary collaboration was relatively uncommon.

*Science–policy–society relations*: Dominant perceptions about the proper relationship between science, policy, and society comprise another dimension that shapes the room for manoeuvre of TDR. This dimension concerns the role attributed to science; the perceived relationship between facts, values, and agency; and freedom of research. These aspects varied greatly across our sites. In Laos, universities are part of the government system and research is expected to contribute evidence for policymaking; freedom of research here is framed by the official policy doctrine. In Bolivia, by contrast, public universities are autonomous, politically very active, and guided by principles of democratization and resistance against dictatorships and imperialism; further, they are often critical of the government. Finally, in Kenya and Madagascar, freedom of research is generally guaranteed, but research results are frequently ignored by policymakers—though this has improved somewhat recently.

*Hierarchies of knowledge holders:* This dimension concerns how particular members of society are ranked in terms of their perceived legitimacy as participants in processes of knowledge production. It also refers to the value attributed to their knowledge as well as the roles they are permitted to assume. In our study sites in Asia and, to a lesser degree, in Africa, we observed rather strong knowledge hierarchies that often reflected the social ranking of different actors. For example, professors or traditional authorities (e.g. wise men/women) are expected to teach, whereas villagers are expected to listen and to learn from them. Both groups—perceived knowledge providers and knowledge receivers—appeared to feel uncomfortable when asked to share their knowledge on an equal footing, as it contradicted their traditional role expectations. In our Latin American study sites, knowledge hierarchies based on people’s social rank were less explicit, though they could be seen between men and women. In all sites, however, deeply rooted colonial relationships between locals and foreigners were an issue.

*Literacy and learning habits:* Literacy refers to the education level of TDR participants, namely their ability to read and write, as well as other variations in the education of different participants. Learning habits concern common modes of learning in both formal education settings and in everyday life. Formal education systems existed in all our case studies, but certain actors—especially older women and members of particular ethnic groups—never went to school or only received a few years of schooling. Many actors were used to learning by means of traditional systems, such as by listening to stories told by elders or by accompanying their parents in their daily work. In this way, “classroom” workshop settings were rather uncommon. At the same time, people’s education levels and learning experiences varied highly across the case studies. Depending on the participants, methods utilizing drawing, acting, gaming, or storytelling worked better than methods requiring a high degree of abstraction and/or literacy.

*Desirability of new knowledge:* This dimension concerns the value attached to creation and sharing of (new) knowledge by different societal actors. It refers to questions about the type(s) of knowledge desired, what knowledge is seen as best kept private, and the perceived role of new knowledge in solving prevailing development challenges. Overall, in all of our case studies, societal actors were primarily interested in context-specific knowledge that was either directly relevant to their day-to-day practices or served their political interests. When these conditions were not met, the societal actors either quickly lost interest, sought to discredit the uncomfortable or undesired knowledge, or actively hindered its generation and distribution. For example, in Laos, it was important to the government that new knowledge conformed to its official policy doctrine, and in Myanmar, all research activity had to be evaluated by the researchers regarding its potential to aggravate latent conflicts. Lastly, local people often viewed solving particular concrete problems—e.g. unsafe drinking water—as more urgent than their own engagement in knowledge co-production. Depending on the context, people frequently welcomed “external” knowledge (e.g. in Madagascar)—though the opposite also occurred (e.g. in Bolivia).

### Exploring paths to transformation

As TDR aims to contribute knowledge for sustainability transformations, exploring paths to transformation is another core part of any TDR process. While impact generation is highly context dependent, we found that the following four elements were consistently important: local actors’ concepts of development and the “good life”, ongoing development dynamics, the level of contestation of given issues, and the level of agency of different actors.

*Concepts of development and the good life:* Concepts of development and the good life involve local people’s ideas about what kind of development is desirable, what it means to lead a good life, and how one can achieve these aims. Improving local economic, material, and social conditions were important development goals mentioned in most of our case study sites (e.g. good houses, children’s education). However, beyond these basic aspects, people’s visions varied substantially within and between case study sites. Differences were often related to distinct spiritual and religious beliefs. For example, according to the Buddhist concept of karma, gaining credits for the next incarnation can be an important motivation for action. Beliefs in spirits and related taboos were also important in several sites, including those related to nature (e.g. forest guardian spirits) or those belonging to one’s ancestors. The importance of these elements is often reflected in the language of local stakeholders, such as in the Lao proverb: “Look backward before moving forward”. Such beliefs sometimes exist side-by-side with other concepts of progress, including desires for mammoth investment projects, which might seem incongruous; other times people’s beliefs represent alternative concepts of development, such as the Bolivian concept of “vivir bien” (including the idea of living well and *in harmony*, as opposed to living *better*), which are seen as contrasting with perceived Western concepts of sustainable development.

*Development dynamics:* These involve ongoing economic, social, and political processes, which shape the options of transformative actions and their prospects of success. In many of our study sites, the macro-political situation was unstable, including instances of political turmoil, unconstitutional changes in government, and social unrest. In some sites, the situation was relatively stable, for example, those sites in Laos where the government has upheld a one-party socialist system for decades. Both situations—unstable vs. stable—displayed their own potentials and limitations for transformative action. In highly volatile situations, new windows of opportunities can quickly emerge, but can also disappear just as swiftly—as experienced in Myanmar, where the transition to a democratic government provided new freedoms, but was suddenly interrupted by a military coup in 2021. In more stable situations, things were more predictable, but fundamental transformations were also less likely. Living conditions were mostly perceived as persistently difficult in all case study sites, however, with highly dynamic economies. In many cases, governments promoted large-scale development projects, which were welcomed by some actors and resisted by others.

*Contestation:* This dimension concerns whether and how the issues tackled by TDR are subject to societal contestation. Contestation can range from different perspectives or heavy tensions between stakeholders all the way to armed conflicts. The issues addressed by our two TDR projects—e.g. land use changes, access to land, water and pasture security, use of agrochemicals—were highly sensitive in all of our case study sites. Different perspectives existed whenever different actors competed for access to land and water—for example, stakeholders focused on biodiversity conservation versus those focused on agricultural production, or stakeholders promoting agroecological production versus those promoting large-scale monocultures. Sometimes these controversies could be approached by means of open dialogue, but other times this was not possible, especially if violence was involved. For example, in Madagascar, tensions between environmentalists and farmers in study sites near national parks were so high that criminal activities became a challenge requiring careful management. Additionally, in Myanmar, questions of land tenure were so sensitive due to the long-running civil war that it was highly delicate to promote any form of transformative action in this setting.

*Actor agency:* This dimension refers to the interest and room for manoeuvre of different actors in terms of contributing to sustainability transformations. Powerful actors with a clear stake in particular issues are especially relevant in fostering or hindering transformative actions. The actor networks and power relations in our case studies were highly complex, with people’s different levels of agency frequently varying depending on the concrete topic. For example, economic actors and the political class were often described as the most capable of inducing change, whereas villagers were described as the most interested, but lacking power. Nevertheless, in several of our sites, the room for manoeuvre of government actors was fairly restricted, despite their legal power, due to their lack of financial resources. In these situations, villagers and other local actors could become quite powerful at the local level and self-organization proved capable of playing a key role in managing rural development processes. In some sites, civil society organizations also attained a considerable amount of agency, such as in Bolivia where syndicates were very effective in mobilizing the masses and fighting for political change, but were less effective at really improving people’s living conditions or environmental conditions.

## Discussion

The contextual conditions investigated in the six case study sites were quite diverse and provided challenges as well as potentials for TDR. Some contextual conditions were revealed to be challenging in all or most of our six cases, suggesting that they are typical for many contexts in the global South (e.g. difficult journeys to reach rural areas, lack of research orientation at universities). Others were very context specific, often varying greatly between the Latin American, Asian, and African settings (e.g. perceptions of the role of science in policymaking, the role of deliberation for opinion formation, and dialogue cultures with different expectations of proper etiquette). However, many of the characteristics point to issues that could be considered just as important in countries of the global North (e.g. the need to become familiar with local dialogue cultures, and consideration of non-academic ways of learning (see Lang et al. 2012; Schneider and Buser 2018).

Several contextual characteristics have been widely mentioned in the TDR literature, for example, by Sim et al. (2019) and Siew et al. (2016) regarding Asia; by Alonzo-Yanez et al. (2019) regarding Latin America; and by Pereira et al. (2020) and van Breda and Swilling (2019) regarding Africa. However, there has been less discussion of contextual characteristics and dimensions related to knowledge co-production, such as accepted practices at universities (Vienni Baptista et al. 2019), and dominant ideas about the proper relationship of science–society–policy (Simon et al. 2020a; Simon 2021). In addition, there is little agreement in the literature regarding how to deal with the challenges arising from them. Recommendations include questioning the social preparedness of certain contexts for TDR (Scholz and Steiner 2015); suggesting alternative methods, approaches, and principles (van Breda and Swilling 2019); decentering academia (Alonso-Yanez et al. 2019); or tailoring concrete suggestions to specific contexts (Sim et al. 2019). For our part, based on the experiences of the case studies presented here, we recommend a nuanced approach involving different coping strategies. These strategies are to be understood as potential ways to address some issues raised and do not necessarily provide solutions in any case.

***Coming together:*** Similar to other studies (Ott and Kiteme 2016; Tejada et al. 2019), we found that contextual conditions prevalent in many global South sites—though not in all—can make it difficult to come together and commence work there. This can turn implementation of TDR into a technically and logistically challenging endeavour (Pereira et al. 2020).

Such practical challenges might be mitigated by adaptive planning and iterative TDR approaches, which make it possible to better respond to known obstacles or sudden changes (Chammas et al. 2020; Ott and Kiteme 2016; Thomas et al. 2018; Simon et al. 2020b). Improving planning and approaches involves making relevant contextual characteristics explicit in research proposals, allocating enough resources and time (Tejada et al. 2019), and flexibilizing corresponding research designs (including systematic risk management, openness to alternatives regarding case selection or methods combination). In very conflictive or fast-changing contexts, this can mean redefining the research approach while it unfolds (van Breda and Swilling 2019).

***Interacting***: The importance of considering the openness for interaction, language issues, and dialogue cultures of diverse groups of actors has also been shown in other studies (Cockburn et al. 2020; Siew et al. 2016; Wang et al. 2019; Woltersdorf et al. 2019). Such consideration is particularly important when foreign researchers are involved (Siew et al. 2016; Wang et al. 2019; Woltersdorf et al. 2019) as well as when national researchers work in multi-ethnic or cross-cultural settings (Alonso-Yanez et al. 2019), especially in cases of long-running conflicts or (interethnic) tensions. According to the literature, intercultural communication appears to be particularly challenging when Western and Asian actors work together, as the former tend to prefer open dialogue and direct communication, whereas the latter are more used to hierarchical working relationships and less-direct communication (Siew et al. 2016; Wang et al. 2019).

Hence, getting acquainted with local contexts and building trust and partnerships might be particularly important in global South contexts (Siew et al. 2016; Thomas et al. 2018; Woltersdorf et al. 2019). For this purpose, long-term partnerships (Wiesmann et al. 2011), collaboration with intermediaries (“joint friends”; see Chammas et al. 2020; Siew et al. 2016), being responsive to local needs, and reciprocal reflexivity proved to be highly valuable in addition to engaging in joint everyday activities.

***Co-producing knowledge:*** Our evaluation revealed fundamental differences between sites in Asia, Latin America, and Africa in terms of all identified contextual conditions influencing co-production of knowledge. We found that some of the related challenges can be tackled by adapting the methodological designs to the dialogue cultures, literacy levels, accepted epistemologies, and knowledge needs of local actors (Ely et al. 2020; Siew et al. 2016; Simon et al. 2020b). This may mean adapting to the preferred ways of joint learning (e.g. storytelling, gaming, drawing, role-playing) or emphasizing joint transformative practices rather than joint reflection alone. In this way, it requires going beyond common practices of abstract scientific theorizing and modelling frequently used in global North approaches to TDR (Alonso-Yanez et al. 2019) as well as viewing TDR not merely as a cognitive process focused on ideal argumentation (Habermas 1981), but as a cognitive–emotional–relational process of social learning (Alonso-Yanez et al. 2019; Boix Mansilla et al. 2016).

But, designing appropriate TDR methods can be challenging when marked differences between social actors are present (e.g. regarding literacy, knowledge needs, or accepted epistemologies; see van Breda und Swilling 2019), when extensive social hierarchies exist between knowledge holders (e.g. lower ranked actors do not speak out, or the knowledge of some actors is not valued by others; see also Siew et al. 2016), or when dominant perceptions of the proper relationship between science, policy, and society serve to constrain open deliberation (Noboa and Upham 2018). Such situations can often only be handled by very experienced facilitators who not only have outstanding moderation and mediation competences, but also the necessary social standing to be respected by the different stakeholders (Noboa and Upham 2018).

However, in other situations, certain contextual characteristics may constrain what we consider core epistemological values of TDR, such as inclusion of all relevant societal actors, articulation of different perspectives, and facilitation of open encounters where participants reflect on the viewpoints of other stakeholders (Pohl and Hirsch Hadorn 2007; van Breda and Swilling 2019). Constraints are sometimes imposed by authoritarian regimes who restrict freedom of expression to the official policy doctrine (Siew et al. 2016), sometimes they are deeply engrained in the structures of society, including research (Ndlovu 2018). As a consequence, researchers and other societal actors might not aim to engage in open encounters to learn together—be it out of fear, conviction or because they never experienced it. What does this mean for the practice of TDR? One might argue that the mentioned epistemological values are strongly related to values of democracy and not necessarily shared by other political systems and schools of thought. From this perspective, insisting would mean to impose external epistemologies, consequently compromising the epistemic foundation of TDR.

However, considering dynamics of power, privilege and bias in the construction of context, knowledge and related epistemologies gives room for a different interpretation. Scholars from Latin America and Africa (e.g. Chouinard and Milley 2016; Ndlovu-Gatsheni 2015) highlight the need to dismantle the power structures involved in dominant, colonial epistemologies because they deprive people, in particular the poor and disadvantaged, of legitimacy and recognition. To enable people to express their own way of life, first, a critical consciousness regarding their own values needs to be created. While the mentioned scholars usually talk about epistemologies imposed by colonization, their considerations might also be applied to other power imbalances producing epistemic entrapment, such as in authoritarian regimes.

Hence, from this perspective, inclusion of all relevant societal actors, and articulation of different perspectives remains a key goal of TDR, but creation of open encounters of joint reflection is only one way of achieving this aim. Alternatively, researchers might begin by working with different groups independently, and strive afterwards to act as neutral mediators, or they might adopt a bottom-up strategy that offers support to disadvantaged people in expressing their voice (Rosendahl et al. 2015; Ott and Kiteme 2016).

***Exploring paths to transformation***: The need for contextualization of TDR is least contested when it comes to exploring paths to transformation (e.g. Cockburn et al. 2020; Pereira et al. 2020; van Breda and Swilling 2019). We found that exploring such paths requires consideration of context-specific concepts of development and “the good life”, as well as reflection on ongoing development dynamics, the degree of contestation of particular issues, and the level of agency of different actors. The combinations of these issues are unique in each context and must be addressed carefully, for example, when formulating research questions, selecting societal actors, or designing transformative practices. In our case studies, several challenging characteristics were relatively common across contexts, including constrained government ability (especially regarding financial resources) to induce change towards sustainability as well as generally unstable macro-political situations (Cockburn et al. 2020; Pereira et al. 2020). Nevertheless, these common constraints do not necessarily call for the same transformative practices in response. For example, some of our case study teams decided to collaborate with government actors expressly to strengthen their capacities, whereas other study teams preferred to work with actors with more perceived agency to induce change (e.g. civil society). However, in all cases, we found it was critical to assess carefully the degree of contestation of particular issues as well as the desirability of new knowledge among different stakeholders. Actors who benefit from the status quo tend to combat the generation and spread of new knowledge, while disadvantaged actors sometimes believe that new knowledge will not make any difference. In addition, researchers must carefully reflect on how to handle “sensitive” knowledge that—when communicated widely—could endanger involved actors. To avoid harms and enhance the potential of TDR in specific contexts, all these aspects should be considered carefully and made explicit in a tentative theory of change that can guide research design and implementation.

## Conclusion

As TDR theories, principles, and methods have largely been conceptualized by researchers in the global North to date, we sought to investigate how contextual characteristics prevalent in global South study sites—in Asia, Latin America, and Africa—impact the design and implementation of TDR. Our results revealed several challenges that should be considered when conducting TDR in these contexts—though many may also be relevant in global North contexts. Common challenges were related to unstable social, political, and environmental conditions, as well as science systems themselves. We argue that TDR requires pragmatic adaptations in these contexts, as well as reflection on underlying epistemological concepts that shape what it means to conduct “good TDR”. To this end, we conclude that TDR must (1) become more adaptive, flexible, and resilient in its design; (2) acknowledge local ways of communicating, researching, and learning in its methods; (3) be embedded in long-term partnerships that facilitate trust building and familiarization with local norms, cultures, and socio-political dynamics; and (4) involve explicit theories of change that reflect local conditions and local ways of working with knowledge. In addition, we advise going beyond the types of abstract scientific theorizing and modelling that characterize TDR in the global North, and instead viewing TDR not only as a cognitive process, but also as a cognitive–emotional–relational process of social learning. At the same time, certain contextual characteristics in the global South may compromise certain core values of TDR, such as inclusion of all relevant societal actors, clear articulation of different perspectives, and creation of open encounters that enable participants to reflect on the perspectives of others. Hindering contextual characteristics include authoritarian governance conditions in which deliberation of competing perspectives is prohibited, as well as settings of open or latent conflict in which the safety of different knowledge holders cannot be guaranteed. In these situations, researchers must reflect on their own epistemological values and perceptions about how to deal with power dynamics in knowledge production while also taking different research cultures into account. One solution is to side with marginalized people to guarantee that their voices are heard. While our research suggests ways forward in these challenging situations, additional studies are needed to investigate how contextual characteristics can be taken into account in an emancipatory way without reproducing power structures.

## Supplementary Information

Below is the link to the electronic supplementary material.

Supplementary file1 (XLSX 33 KB)
